# The effects of preoperative intestinal dysbacteriosis on postoperative recovery in colorectal cancer surgery: a prospective cohort study

**DOI:** 10.1186/s12876-021-02035-6

**Published:** 2021-11-25

**Authors:** Yuwei Liu, Wanbin He, Jie Yang, Yuhua He, Ziqiang Wang, Ka Li

**Affiliations:** 1 West China School of Nursing, Sichuan University/Department of Gastrointestinal Surgery, West China Hospital, Sichuan University, Chengdu, People’s Republic of China; 2 Nursing Key Laboratory of Sichuan Province, Chengdu, People’s Republic of China

**Keywords:** Colorectal cancer, Intestinal dysbacteriosis, Diarrhoea, Infectious complications, Anastomotic leakage

## Abstract

**Background:**

Accumulating evidence suggests a critical role of intestinal dysbacteriosis in surgical site infections and anastomotic leakage after abdominal surgery. However, a direct correlation between pre-existing dysbacteriosis and postoperative infectious complications has not yet been established clinically.

**Methods:**

A total of 353 consecutive patients who underwent colorectal cancer (CRC) surgery were enrolled. Gram-stained faecal smears were tested at admission and the first defecation after surgery. Intestinal dysbacteriosis was graded into three groups: normal or slightly decreased intestinal microflora (grade 1), moderate dysbacteriosis (grade 2), and severe dysbacteriosis (grade 3). Clinical outcomes were postoperative infections and anastomotic leakage within 30 days after surgery.

**Results:**

At the preoperative assessment, 268 (75.9%) patients had normal or slightly decreased intestinal microflora, 58 (16.4%) patients had moderate dysbacteriosis, and 27 (7.6%) patients had severe dysbacteriosis. The patients with preoperative dysbacteriosis had a higher rate of early postoperative diarrhoea (grade 2: OR = 4.53, 95% CI 2.28–9.00, grade 3: OR = 4.52, 95% CI 1.81–11.31), total complications (grade 3 40.7% vs. grade 2 25.9% vs*.* grade 1 12.7%, *P* < 0.001), and anastomotic leakage (grade 3 11.1% vs. grade 2 5.2% vs*.* grade 1 1.5%, *P* = 0.002). An interaction effect among preoperative dysbacteriosis and early postoperative diarrhoea on total complications was observed in rectal cancer patients (*P* for interaction = 0.007).

**Conclusions:**

An imbalance of the intestinal microbiome exists in a considerable proportion of CRC patients before surgery. Preoperative dysbacteriosis is associated with higher rates of early postoperative diarrhoea, which further correlates with infectious complications and anastomotic leakage. However, the contribution of preoperative dysbacteriosis to the occurrence of anastomotic leakage needs to be clarified in further studies.

*Trial registration* ChiCTR, ChiCTR1800018755. Registered 8 October 2018—Retrospectively registered, http://www.chictr.org.cn/ChiCTR1800018755.

**Supplementary Information:**

The online version contains supplementary material available at 10.1186/s12876-021-02035-6.

## Background

The past decades have witnessed advances in colorectal surgical techniques and perioperative management such as enhanced recovery after surgery (ERAS) programmes. However, surgical infectious complications are still frequently observed among colorectal cancer (CRC) patients, with an overall incidence of 18.9–46% [1–3]. Anastomotic leakage remains one of the most severe complications after surgery [4], leading to prolonged hospitalization [5], compromised quality of life, and increased mortality [6]. Moreover, these complications were associated with increased healthcare expenditure [7].

The gut microbiota has been shown to play a critical role in postoperative recovery. The human gastrointestinal tract is colonized by thousands of species of bacteria [8]. These microbial communities perform multiple functions for human health, such as competitively preventing pathogens, stimulating local immunity, preserving mucosal barrier function, reducing the inflammatory response, and synthesizing nutrients [9, 10]. Segawa et al. [11] reported that intestinal *Lactobacillus* could produce polyphosphate, which helps to maintain the mucosal barrier and to regulate the inflammatory response. Ample evidence indicates that disrupting the balance of intestinal flora contributes to septic complications through endogenous enteral bacterial translocation. Ralls et al. [12] found that a low level of enteric microbial diversity correlated strongly with a higher incidence of postoperative infections and anastomotic disruption in paediatric and adult patients who underwent small intestinal resection. Animal studies demonstrated that *Pseudomonas aeruginosa* [13] and *Enterococcus fecalis* [14], commensal bacteria of the intestine, could transform into tissue destroying phenotypes, and cause anastomotic leakage by degrading collagen at the anastomosis.

Most of the studies focused on how postoperative intestinal dysbacteriosis involves surgical complications [15]. However, the abundance and diversity of the gut microbiome in CRC patients may have already been changed even before admission. It was reported that a reduction in butyrate producers and an increase in opportunistic pathogens, such as *Enterococcus*, *Escherichia*, *Shigella*, *Klebsiella*, *Streptococcus,* and *Peptostreptococcus*, constitute a major structural imbalance of gut microbiota in CRC patients [16]. Colorectal surgery, carrying the highest risk of surgical site infection, is most likely to be impacted by pre-existing intestinal dysbacteriosis. Several studies have revealed that the use of synbiotics for 7 days preoperatively is associated with reductions in morbidity in CRC patients [17, 18]. However, the loss of preoperative intestinal microbial diversity has not yet been directly identified as a potential risk factor for outcomes after surgery. We speculated that intestinal dysbacteriosis may already exist at patient admission and further affects patients’ recovery after surgery. Therefore, a prospective cohort study was conducted to investigate the correlation between the patterns of intestinal microbiota before surgery and the postoperative complications in CRC patients.

## Methods

### Patients

From March 2016 to April 2019, patients with biopsy confirmed CRC at the Department of Gastrointestinal Surgery, West China Hospital of Sichuan University, were enrolled in this study. The inclusion criteria were as follows: (1) age 18–90 years, (2) first primary invasive malignant CRC, identified through the admitting diagnosis from the electronic medical records (EMRs) system, which could be further confirmed by discharging diagnosis with International Classification of Diseases coding (ICD-10, C18, C19, C20), (3) scheduled CRC radical resection for the first time, (4) absence of inflammatory bowel disease (IBD) or intestinal stoma before surgery, and (5) absence of active tumours at other sites, either concurrent primary tumour or previously treated tumours. Pathologic diagnosis of CRC was made based on biopsy under colonoscopy at the outpatient department and confirmed after radical resection. Patients were excluded if they underwent emergent surgery, local excision of the tumour, or diverting stoma surgery.

**Fig. 1:**
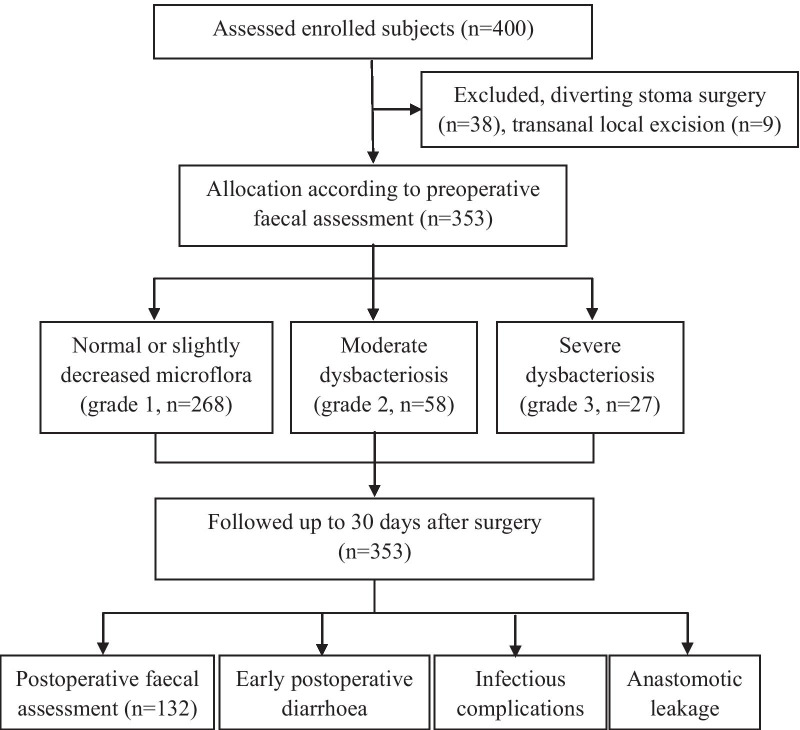
Flow diagram of patient recruitment. A total of 400 patients were initially selected for inclusion. In total, 353 patients were enrolled and their outcomes were tracked up to 30 days after surgery

A total of 400 patients who underwent colon or rectal cancer resection were enrolled, 38 patients were excluded for diverting stoma surgery, and 9 patients were excluded for transanal local excision. Figure 1 shows the flow diagram of patient recruitment in this study. The study protocols were approved by the ethics committee of Sichuan University, and written informed consent for participation was obtained from the patients before enrolment in the study. The clinical trial registration number is ChiCTR1800018755. This manuscript has been reported in accordance with the STROBE guidelines [19].

### Study design and patient care

This is a prospective observational cohort design. For eligible patients, faecal samples were collected both at admission and at the first defecation after surgery. Patients were classified into three groups according to the preoperative faecal examination results: normal or slightly decreased intestinal microflora (grade 1), moderate intestinal dysbacteriosis (grade 2), and severe intestinal dysbacteriosis (grade 3). All patients were managed by following the recommendations of the Enhanced Recovery After Surgery (ERAS®) Society [20]. On the day before surgery, left colon and rectal cancer patients received mechanical bowel preparation with an oral solution prepared with 69 g polyethylene glycol electrolyte powder in 2000 ml of water. All patients received prophylactic antibiotics (cefoxitin or cefmetazole, 2 g, Q8h) intravenously from 30 min before surgery that were maintained for 48 h postoperatively. Prolonged or additional antibiotic therapy was prescribed when patients developed postoperative infectious complications.

### Faecal assays

**Fig. 2:**
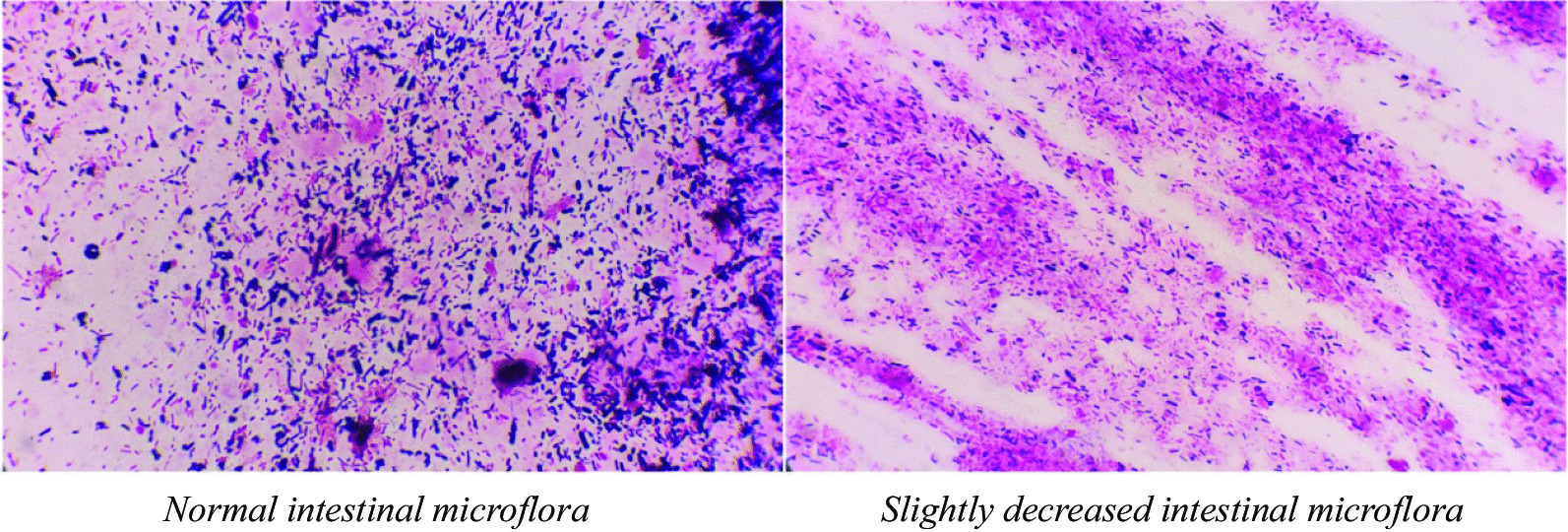
Normal or slightly decreased intestinal microflora (grade 1), normal intestinal microflora with abundant bacteria of various morphologies covering the field (left), or a slight decrease in the total bacterial population (right)

**Fig. 3:**
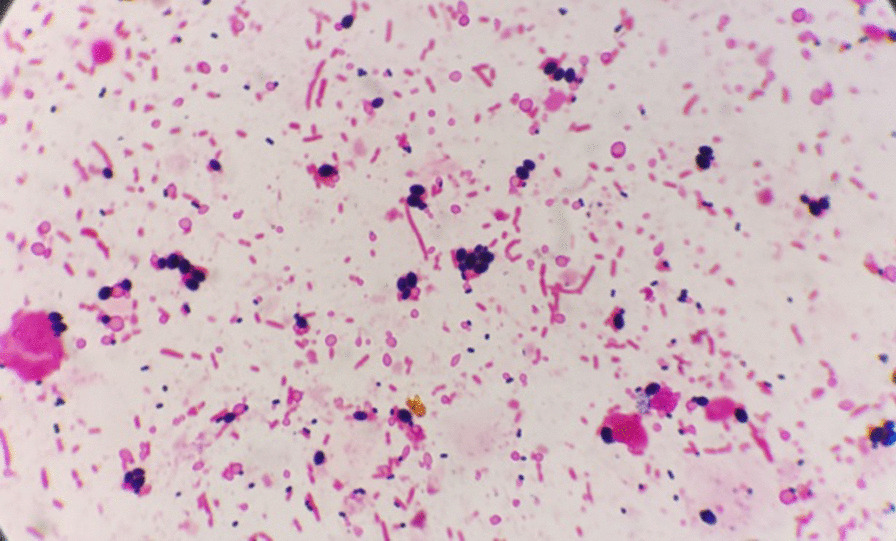
Moderate dysbacteriosis (Grade 2), with a significant reduction in bacterial population, and only one or two kinds of gram-stained microflora predominating the field

**Fig. 4:**
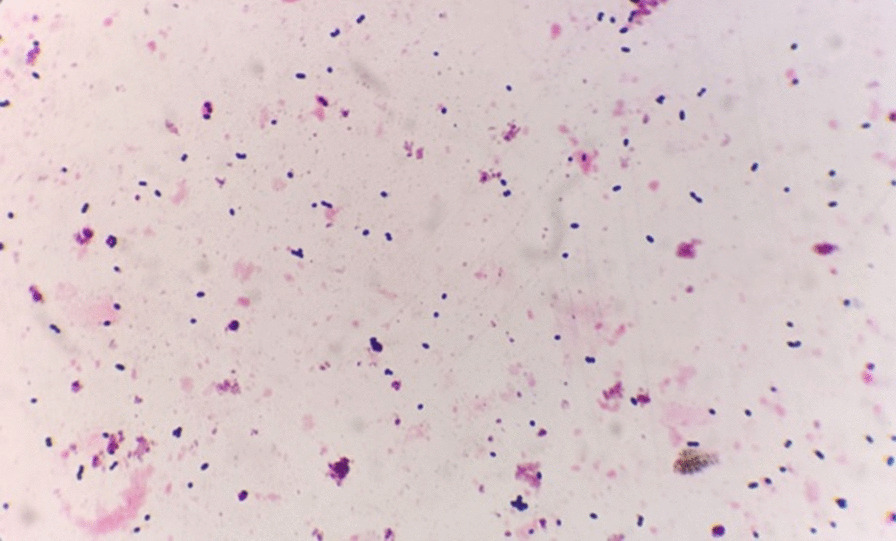
Severe dysbacteriosis (Grade 3), with depleted bacterial population

Under nurses’ instructions, 3 g of faeces was collected from the centre of the stool, kept in a sterile container, and sent within 30 min for faecal smear testing at the laboratory centre affiliated with our hospital. The microflora was tested with gram staining and classified as gram-positive bacillus, gram-negative bacillus, and gram-positive streptococcus. The severity of dysbacteriosis was graded, according to the total bacterial population and imbalanced structure of the faecal flora:

- Normal intestinal microflora, defined as abundant bacteria of various morphologies covering the whole field, is shown in Fig. 2.
- Slightly decreased intestinal microflora was defined as a slight decrease in the total bacterial population or one kind of gram-stained flora (Fig. 2). Considering that the intestinal microflora may fluctuate physiologically daily [21], normal or slightly decreased intestinal microflora were classified as grade 1 in this study.
- Moderate dysbacteriosis (grade 2), defined as a significant reduction in the bacterial population with one kind of gram-stained microflora predominating the field, or a reversed ratio of bacillus to coccus (≤ 1:1), is shown in Fig. 3. The ratio of Gram-negative to Gram-positive prokaryotes varies widely in different people [22]. In general, bacilli are much more common than cocci, with a ratio of approximately 3:1 (73.5–97.5% vs. 2.5–26.5%) in healthy adults [23].
- Severe dysbacteriosis (grade 3), defined as depletion of the bacterial population or the presence of pathogenic bacteria of distinctive morphology (Fig. 4).

All tests and reports were executed by specialized microbiologists from the clinical laboratory of West China Hospital of Sichuan University. Additionally, to evaluate the stability of the stool gram stain examination, we consecutively collected 40 stool samples from 20 patients for testing. Two specimens from the same person at the same time were examined by different microbiologists in the laboratory, and the result of the other specimen remained unknown to the microbiologist.

### Clinical variables extracted for analysis

The patient demographic and clinical characteristics were recorded, including age, sex, tumour location, body mass index (BMI), neoadjuvant therapy, mechanical bowel preparation, the tumour-node-metastasis (TNM) stage, American Society of Anaesthesiologists (ASA) score, detailed surgical information, perioperative antibiotic usage and results of laboratory tests. The diagnosis of preoperative comorbidities was made through systematic patient history review and preoperative examinations by specialist colorectal surgeons, which can be identified through admitting diagnosis in the EMRs. Data were prospectively recorded using a case report form (CRF) constructed specifically for this study.

### Postoperative observations and definition of complications

Clinical outcomes were total or surgical postoperative complications within 30 days after surgery. Total complications included pulmonary infections, urinary tract infections, fever of unknown origin (≥ 38.5 °C), and surgical complications. Surgical complications included surgical site infections (SSIs) and anastomotic leakage. SSIs included surgical incision infections, abdominal infections, and pelvic infections.

Infectious complications were described in accordance with the Centres for Disease Control Definitions [24]. Patients with mild fever (≥ 37 °C) before discharge or prolonged ileus or other unusual abdominal complaints received chest and abdominal computed tomography (CT) scans for evaluation of leakage, intraperitoneal infection, or pulmonary infection. Anastomotic leakage was defined according to the proposal by the International Study Group of Rectal Cancer [25]. CT with contrast enema was used in patients with a suspicious diagnosis of anastomotic leakage. A local collection of fluid with gas around the anastomosis was confirmed as leakage. If a patient had simultaneous infections of the same body cavity, for instance, anastomotic leakage and pelvic abscess in the proximity of the anastomosis, only one case was counted into surgical or total postoperative complications.

Postoperative symptoms or clinical features, such as early postoperative diarrhoea [26], and hypoalbuminemia [27], that might be associated with outcomes were also recorded. Early postoperative diarrhoea was defined as three or more unusually loose or liquid stools per day [28], occurring within 7 days after surgery. Postoperative hypoalbuminemia was defined as albumin < 30 g/L [27] within 3 days after surgery. The postoperative length of hospital stay was calculated from the day of surgery to discharge. Patients were followed up through the outpatient clinic at 2 weeks after discharge and received a telephone follow-up again at 30 days after surgery to track their outcomes. All enrolled patients completed the follow-up procedure.

### Statistical analysis

SPSS version 26.0 (SPSS, Chicago, IL, USA) was used for statistical analysis. One-way ANOVA was used for comparison of normally distributed quantitative data, and the Kruskal–Wallis test was used for comparison of non-normally distributed quantitative data. Comparison of categorical data was conducted with the Chi-square test or the Kruskal–Wallis test for ordered categorical data. Spearman's rank-order correlation test was used to analyse the relationship between preoperative and postoperative dysbacteriosis. The Chi-square test for trend was used to assess whether the association between the preoperative dysbacteriosis and postoperative outcomes follows a trend. Univariate logistic regression analysis was undertaken to assess factors influencing early postoperative diarrhoea or total postoperative complications. Variables in the univariate logistic regression analysis that had a significance level of *P* < 0.1 were included in multivariate logistic regression. *P* < 0.05 was considered statistically significant, and the significance level of pairwise comparisons was calibrated according to the Bonferroni method.

## Results

### Patient characteristics

In total, 353 patients were retained for the analysis (Fig. 1). The average age of the enrolled patients was 60.7 ± 12.6 years (over the range of 21–88 years), 150 (42.5%) were male, and 203 (57.5%) were female. Among these patients, 204 (57.8%) had rectal cancer, and 149 (42.2%) had colon cancer, and 77 (21.8%) received neoadjuvant therapy before admission. The detailed demographic and clinical characteristics are listed in Table 1.

**Table 1:** Characteristics of the patients (N = 353)

Variable	Total (n = 353)	Dysbacteriosis grade 1 (n = 268)	Dysbacteriosis grade 2 (n = 58)	Dysbacteriosis grade 3 (n = 27)	F/*x*^2^	*P*
Age (years), Mean ± SD (range)	60.7 ± 12.6 (21–88)	60.5 ± 12.9 (21–88)	61.4 ± 12.1 (31–86)	60.7 ± 10.9 (43–79)	0.120	0.887
Sex, N(%)					2.124	0.351
Male	150 (42.5%)	110 (41.0%)	25 (43.1%)	15 (55.6%)		
Female	203 (57.5%)	158 (59.0%)	33 (56.9%)	12 (44.4%)		
Tumour location, N(%)					7.282	0.026
Rectum	204 (57.8%)	160 (59.7%)	25 (43.1%)	19 (70.4%)		
Colon	149 (42.2%)	108 (40.3%)	33 (56.9%)	8 (29.6%)		
BMI (kg/m^2^)^†^, N(%)					3.524	0.710^§^
Underweight (< 18.5)	24 (6.8%)	16 (6.0%)	5 (8.6%)	3 (11.1%)		
Normal (18.5–24.9)	246 (69.7%)	184 (68.7%)	43 (74.1%)	19 (70.4%)		
Overweight (25.0–29.9)	80 (22.7%)	65 (24.3%)	10 (17.2%)	5 (18.5%)		
Obese (≥ 30.0)	3 (0.8%)	3 (1.1%)	0 (0.0%)	0 (0.0%)		
Comorbidities^‡^, N(%)						
Cardiopulmonary disease	39 (11.0%)	34 (12.7%)	3 (5.2%)	2 (7.4%)	3.134	0.209
Diabetes	26 (7.4%)	19 (7.1%)	4 (6.9%)	3 (11.1%)	0.604	0.739
Liver or kidney disease	12 (3.4%)	9 (3.4%)	1 (1.7%)	2 (7.4%)	1.913	0.409^§^
Preoperative anaemia	136 (38.5%)	96 (35.8%)	26 (44.8%)	14 (51.9%)	3.825	0.146
Preoperative albumin (g/L), Mean ± SD	42.39 ± 4.97	42.63 ± 4.66	42.18 ± 6.14	40.44 ± 4.95	2.436	0.089
Neoadjuvant therapy, N(%)	77 (21.8%)	65 (24.3%)	7 (12.1%)	5 (18.5%)	4.337	0.111
ASA score, N(%)					8.330	0.198 ^§^
1	7 (2.0%)	3 (1.1%)	3 (5.2%)	1 (3.7%)		
2	271 (76.8%)	211 (78.7%)	42 (72.4%)	18 (66.7%)		
3	74 (21.0%)	53 (19.8%)	13 (22.4%)	8 (29.6%)		
4	1 (0.3%)	1 (0.4%)	0	0		
TNM stage, N(%)					8.295	0.217
I	67 (19.0%)	48 (17.9%)	14 (24.1%)	5 (18.5%)		
II	118 (33.4%)	86 (32.1%)	23 (39.7%)	9 (33.3%)		
III	147 (41.6%)	120 (44.8%)	15 (25.9%)	12 (44.4%)		
IV	21 (5.9%)	14 (5.2%)	6 (10.3%)	1 (3.7%)		
Surgical approach, N(%)					1.203	0.548
Open abdominal	86 (24.4%)	69 (25.7%)	12 (20.7%)	5 (18.5%)		
Laparoscopic	267 (75.6%)	199 (74.3%)	46 (79.3%)	22 (81.5%)		
Operation time, N(%)					0.844	0.656
< 3 h	100 (28.3%)	79 (29.5%)	15 (25.9%)	6 (22.2%)		
≥ 3 h	253 (71.7%)	189 (70.5%)	43 (74.1%)	21 (77.8%)		
Intraoperative blood loss (ml), Median (IQR)	50 (30–80)	50 (30–80)	50 (30–60)	50 (30–100)	2.639	0.267
Mechanical bowel preparation, N(%)	214 (60.6%)	162 (60.4%)	33 (56.9%)	19 (70.4%)	1.415	0.493

Abbreviations: *BMI* body mass index. *ASA* American Society of Anaesthesiologists. *TNM* the tumour-node-metastasis staging

^†^BMI was classified according to the World Health Organization criteria

^‡^Cardiopulmonary diseases including chronic bronchitis, bronchiectasis, chronic obstructive pulmonary disease, and chronic pulmonary heart disease. Liver or kidney disease includes hepatic cirrhosis and chronic renal dysfunction. Preoperative anaemia was defined as haemoglobin (Hb) < 120 g/L for women and < 135 g/L for men

^§^The *P* value was obtained by Fisher's exact test

### Assessment of the stability of stool gram stain examinations

A total of 40 faecal samples (from 20 patients) were collected and sent for laboratory tests, to evaluate the stability of the stool gram stain examination. Among these samples, the results of 18 pairs of specimens were consistent, but two pairs of samples were assessed as grade 1 and grade 2. There may be a slight bias in the subjective classification judgement, but the consistency of two stool smears in the diagnosis of dysbacteriosis reached an acceptable level of 90% (18/20), with a kappa coefficient of 0.806 (*P* < 0.001).

### Correlation between preoperative and postoperative dysbacteriosis

Prior to surgery, 181 (51.3%) patients had normal intestinal microflora, 87 (24.6%) patients had slightly decreased intestinal microflora, 58 (16.4%) patients had moderate dysbacteriosis, and 27 (7.6%) had severe dysbacteriosis (Table 1). Among the 353 patients, 132 (37.4%) patients had stool smears after surgery, with a median length of postoperative stay of 6.0 (interquartile range, IQR 5.0–8.0) days. The others had no defecation or failed to collect enough qualified faecal specimens before discharge, with a median postoperative stay length of 5.0 (IQR 4.0–6.5) days; hence, they had no qualified faecal smear evaluations. Spearman's rank-order correlation test was conducted for these 132 pairs of faecal samples. There was a moderate, but significant, correlation between preoperative and postoperative dysbacteriosis (Spearman *r*_*s*_ = 0.402, gamma *G* = 0.602, *P* < 0.001). Among 73 patients who had grade 2/3 dysbacteriosis after surgery, 47.9% (35/73) had dysbacteriosis before surgery (*P* < 0.001), as shown in Table 2.

**Table 2:** Intestinal dysbacteriosis before and after the operation (N = 132)

Preoperative dysbacteriosis	Postoperative dysbacteriosis				*r*_*s*_	*P*
	Grade 1	Grade 2	Grade 3	Sum		
Grade 1	52 (57.8%)	25 (27.8%)	13 (14.4%)	90	0.402	< 0.001
Grade 2	3 (11.5%)	16 (61.5%)	7 (26.9%)	26		
Grade 3	4 (25.0%)	2 (12.5%)	10 (62.5%)	16		
Sum	59	43	30	132		

The *P* value was obtained by Spearman's rank-order correlation

### Correlation of the preoperative dysbacteriosis and short-term outcomes

Early postoperative diarrhoea occurred in 21.5% (76/353) of patients. A total of 52 patients underwent CT examination for abdominal symptoms after surgery, and 10 of them were diagnosed with anastomotic leakage. A sum of 53 patients developed 60 cases of postoperative infectious complications. No death cases happened in our study. Compared to patients with preoperative dysbacteriosis grade 1, those with preoperative dysbacteriosis grade 2 or grade 3 developed more total complications (grade 3 40.7% vs. grade 2 25.9% vs. grade 1 12.7%, *P* < 0.001). A statistically significant linear trend for increasing early postoperative diarrhoea, surgical complications, anastomotic leakage, pulmonary infections, and total complications as the grade of preoperative dysbacteriosis increased was observed (Chi-square test for trend: *P* < 0.05). Regarding incision infections, urinary tract infections, and fever of unknown origin, no significant differences between groups were observed, as shown in Table 3.

**Table 3:** Postoperative short-term outcomes of each group (N = 353)

Postoperative short-term outcomes	Preoperative intestinal dysbacteriosis			Sum	*x*^2^/*H*	*P*
	Grade 1 (n = 268)	Grade 2 (n = 58)	Grade 3 (n = 27)			
Early postoperative diarrhoea	38 (14.2%)	25 (43.1%)	13 (48.1%)	76 (21.5%)	32.704	< 0.001^†^
Surgical complications	13 (4.9%)	6 (10.3%)	5 (18.5%)	24 (6.8%)	8.481	0.004^†^
Incision infections	5 (1.9%)	1 (1.7%)	2 (7.4%)	8 (2.3%)	2.091	0.148^†^
Abdominal/Pelvic infections	5 (1.9%)	2 (3.4%)	3 (11.1%)	10 (2.8%)	6.470	0.011^†^
Anastomotic leakage	4 (1.5%)	3 (5.2%)	3 (11.1%)	10 (2.8%)	9.429	0.002^†^
Pulmonary infections	16 (6.0%)	7 (12.1%)	6 (22.2%)	29 (8.2%)	9.733	0.002^†^
Urinary tract infections	3 (1.1%)	1 (1.7%)	0 (0.0%)	4 (1.1%)	0.049	0.824^†^
Fever of unknown origin	2 (0.7%)	1 (1.7%)	0 (0.0%)	3 (0.8%)	0.002	0.963^†^
Total postoperative complications	34 (12.7%)	15 (25.9%)	11 (40.7%)	60 (17.0%)	17.481	< 0.001^†^
Length of postoperative stay (days)	6.0 (3–32)	6.0 (3–32)	6.0 (3–31)	6.0 (3–16)	1.867	0.393
Length of antibiotics therapy (days)	2.0 (1–24)	2.0 (1–24)	3.0 (2–20)	2.0 (1–18)	9.791	0.007^‡^
Antibiotics regimen^§^, n(%)						
Cefoxitin or Cefmetazole	260 (97.0%)	54 (93.1%)	22 (81.5%)	336 (95.2%)	21.163	0.002
Cefoperazone/sulbactam (Sulperazon)	3 (1.1%)	1 (1.7%)	3 (11.1%)	7 (2.0%)		
Piperacillin/tazobactam (Tazocin)	2 (0.7%)	2 (3.4%)	2 (7.4%)	6 (1.7%)		
Moxifloxacin (Avelox)	3 (1.1%)	1 (1.7%)	0 (0.0%)	4 (1.1%)		

The data are presented as n(%) for categorical variables or the median (range) for continuous variables

^†^The *P* value was obtained by Chi-square test for trend

^‡^Pairwise multiple comparisons after Kruskal–Wallis test: grade 1 vs. grade 3, adjusted *P* < 0.05

^§^Antibiotic regimen: cefoxitin or cefmetazole, 2.0 g Q8h, Sulperazon 3.0 g Q8h, tazoxin 4.5 g Q8h, avelox 0.4 g Qd

### Risk factors for total postoperative complications

Logistic regression was conducted to identify risk factors for total postoperative complications. The following variables were found to be associated with total complications in univariate analysis: preoperative dysbacteriosis, early postoperative diarrhoea, postoperative hypoalbuminemia, intraoperative blood loss, and sex (*P* < 0.1, Additional file 1: Table 1). They were included for multivariate logistic regression. As to early postoperative diahhroea, only those that occurred before postoperative complications were included in the logistic regression. Finally, preoperative dysbacteriosis grade 3 (OR = 3.24, 95% CI 1.21–8.62, *P* = 0.019), early postoperative diarrhoea (OR = 2.89, 95% CI 1.43–5.81, *P* = 0.003), and hypoalbuminemia (OR = 2.95, 95% CI 1.24–7.02, *P* = 0.014) were indicated as independent risk factors for total complications, as shown in Table 4.

**Table 4:** Logistic regression of total postoperative complications

Parameter	Univariate logistic regression		Multivariate logistic regression^§^	
	OR (95% CI)	*P*	Adjusted OR (95% CI)	*P*
*Total patients*				
Preoperative dysbacteriosis^†^				
Grade 2	2.26 (1.07–4.77)	0.033	1.64 (0.73–3.70)	0.232
Grade 3	4.23 (1.74–10.32)	0.002	3.24 (1.21–8.62)	0.019
Early postoperative diarrhoea^‡^	3.66 (1.93–6.95)	0.001	2.89 (1.43–5.81)	0.003
Postoperative hypoalbuminema	2.71 (1.25–5.86)	0.012	2.95 (1.24–7.02)	0.014
Intraoperative blood loss (≥ 100 ml)	1.80 (0.92–3.53)	0.085	1.19 (0.57–2.48)	0.643
Sex (Female)	1.81 (0.94–3.45)	0.074	2.35 (1.15–4.78)	0.019
*Colon cancer*				
Preoperative dysbacteriosis^†^				
Grade 2	1.76 (0.60–5.13)	0.300	2.01 (0.63–6.39)	0.238
Grade 3	1.13 (0.13–10.00)	0.912	1.24 (0.13–12.24)	0.855
Early postoperative diarrhoea	2.37 (0.81–6.95)	0.115	1.71 (0.55–5.33)	0.358
BMI (≥ 25.0 kg/m^2^)	3.54 (1.28–9.81)	0.015	3.72 (1.27–10.92)	0.017
*Rectal cancer*				
Preoperative dysbacteriosis^†^				
Grade 2	2.96 (1.03–8.53)	0.045	1.62 (0.50–5.26)	0.423
Grade 3	6.46 (2.27–18.39)	< 0.001	3.64 (1.16–11.47)	0.027
Postoperative hypoalbuminema	3.04 (1.05–8.76)	0.040	2.15 (0.67–6.83)	0.196
Early postoperative diarrhoea	4.70 (2.08–10.64)	< 0.001	3.17 (1.27–7.94)	0.014

^†^Reference group: Grade 1

^‡^Sum to 4 cases of diarrhoea that happened after postoperative pulmonary infections were not included in the logistic regression

^§^Hosmer–Lemeshow test: total patients x^2^= 3.220, *P* = 0.666, colon cancer x^2^ = 0.889, *P* = 0.926, rectal cancer x^2^= 3.119, *P* = 0.374

### Risk factors for early postoperative diarrhoea

Since our results showed that early postoperative diarrhoea was a risk factor for predicting postoperative complications, we further investigated the correlation between preoperative dysbacteriosis and early postoperative diarrhoea. Patients with preoperative dysbacteriosis grade 2 or grade 3 suffered higher rates of early postoperative diarrhoea than those with grade 1 (grade 3 48.1% vs. grade 2 43.1% vs. grade 1 14.2%, *P* < 0.001). Taking variables that had a significant level of *P* < 0.1 (Additional file 1: Table 2) into multivariate logistic regression analysis, it was shown that patients with grade 2 or grade 3 dysbacteriosis had a higher risk of postoperative diarrhoea than those with grade 1 (grade 2: OR = 4.53, 95% CI 2.28–9.00, grade 3: OR = 4.52, 95% CI 1.81–11.31), as shown in Table 5.

**Table 5:** Logistic regression of postoperative diarrhoea

Exposure	Univariate logistic regression		Multivariate logistic regression^‡^	
	OR (95%CI)	*P*	Adjusted OR (95%CI)	*P*
*Preoperative dysbacteriosis*^†^				
Grade 2	4.59 (2.46–8.55)	< 0.001	4.53 (2.28–9.00)	< 0.001
Grade 3	5.62 (2.45–12.88)	< 0.001	4.52 (1.81–11.31)	0.001
Surgical approach (laparoscopic)	1.94 (0.99–3.81)	0.052	2.30 (1.07–4.93)	0.033
BMI (≥ 25.0 kg/m^2^)	1.85 (1.06–3.24)	0.031	2.62 (1.37–5.00)	0.004
Intraoperative blood loss (≥ 100 ml)	2.00 (1.13–3.53)	0.017	1.69 (0.87–3.28)	0.122
Length of antibiotics therapy > 3 days	4.33 (2.42–7.75)	< 0.001	4.30 (2.21–8.39)	< 0.001

^†^Reference group: Grade 1

^‡^Hosmer–Lemeshow test x^2^= 3.574, *P* = 0.734

Abbreviations: *BMI* body mass index, *OR* odds ratio, *95 CI* 95 confidence interval

### Subgroup analysis of colon and rectal cancer

Patients with tumours at different sites had significantly different demographic and clinical characteristics in terms of BMI, TNM stage, neoadjuvant therapy, bowel preparation, and length of operation (Additional file 1: Table 3). Therefore, we performed the subgroup analysis. More rectal cancer patients were identified with severe preoperative dysbacteriosis (rectum group 9.3% vs. colon group 5.4%), while colon cancer patients showed more moderate preoperative dysbacteriosis (rectum group 12.3% vs. colon group 22.1%), Chi-square test *P* = 0.026 (Additional file 1: Table 3). According to multivariate logistic regression analyses, the correlation between preoperative dysbacteriosis and early postoperative diarrhoea was identified in both patient cohorts undergoing colon or rectal resections (Additional file 1: Table 4). However, early postoperative diarrhoea was only identified as a risk factor for postoperative complications in the rectum group (Table 4).

### Interaction analysis

Further, we explored a possible interaction effect among preoperative dysbacteriosis and early postoperative diarrhoea on total complications by using multiple logistic regression analysis (multiplicative method). The interaction was observed in rectal cancer patients (*P* for interaction = 0.007), but not in colon patients (*P* for interaction = 0.524), as shown in Table 6.

**Table 6:** Combined effect of preoperative dysbacteriosis and early postoperative diarrhoea on total postoperative complications

Variable	Total patients		Colon cancer		Rectal cancer	
	Adjusted OR^†^ (95% CI)	*P* for interaction	Adjusted OR^‡^ (95% CI)	*P* for interaction	Adjusted OR^§^ (95% CI)	*P* for interaction
Preoperative dysbacteriosis		0.001		0.524		0.007
Grade 1× diarrhoea	1 (reference)		1 (reference)		1 (reference)	
Grade 2× diarrhea	0.98 (0.27–3.57)	0.975	0.83 (0.10–7.13)	0.866	1.05 (0.21–5.31)	0.949
Grade 3× diarrhoea	10.14 (2.93–35.05)	< 0.001	5.37 (0.28–102.46)	0.264	7.97 (2.20–28.92)	0.002

Multivariate logistic regression: ^†^Adjusted for postoperative hypoalbuminema, intraoperative blood loss, and sex. ^‡^Adjusted for BMI. ^§^Adjusted for postoperative hypoalbuminema

## Discussion

The gastrointestinal tract and the diversity in the microbiome compose a complex ecosystem. However, this ecosystem coexists in a fragile balance and is vulnerable to disturbance [29]. The preoperative manipulation of the gut microbiota is an interesting alternative to prevent infectious complications after surgery [18]. To the best of our knowledge, this is the first study to shed light on the association between preoperative stool dysbacteriosis and postoperative infectious outcomes.

Our work showed that an obvious imbalance of the intestinal microbiome occurs in nearly a quarter of CRC patients before surgery. Most of these patients had a reduced bacterial population and lack of diversity in microbiota. Some patients were even depleted of bacteria before surgery. Multiple factors, including colorectal tumour itself, previous comorbid diseases, self-medication, diet and environment, were possible causes of the microflora`s dramatic change. Altered host-microbiota interactions and dysbiosis have been involved in the oncogenesis of CRC [30]. Specific pathogens, for example, *Fusobacterium nucleatum*, has been identified as a pro-carcinogenic bacterium in various stages of CRC [31]. Cytolethal distending toxin (CDT), produced by gram-negative bacteria, is by far the most well-characterized genotoxin. Several microorganisms relevant to colorectal, gastric, and gallbladder cancer (such as *E. coli, Helicobacter spp.* and *S. Typhi*) are all CDT producers [30]. Colonoscopy is necessary for the diagnosis of CRC before admission, but mechanical bowel preparation induces a significant reduction in *Lactobacillaceae* and an increase in *Enterobacteriaceae* abundance [32]. Neoadjuvant chemo- or chemoradiotherapy may also contribute to preoperative dysbacteriosis. Chemotherapy for non-Hodgkin lymphoma can lead to an increase in the abundance of *Proteobacteria* and a substantial decrease in *Firmicutes* and *Actinobacteria* [33]. In our study, the median time between colonoscopy at the outpatient clinic (or the latest neoadjuvant therapy) and fecal assay was 12 days (IQR, 8–28 days). How did intestinal microbiota changed and recovered after these interventions remain unknown. Moreover, the laboratory of public health safety of FuDan University of China detected 18 kinds of antibiotics from 47.8% of children's urine samples, with concentrations of 0.1–20 ng/mL, suggesting general exposure to low-dose antibiotics from the diet and environment [34]. The wide use of antibiotics in livestock industry [35], improper use of food additives, iatrogenic intake of antibiotics [36], etc., are unneglectable factors associated with an increased prevalence of intestinal dysbacteriosis.

Preoperatively existing dysbacteriosis may lead to adverse postoperative outcomes. Our study found that, compared to patients with normal microbiota, those with dysbacteriosis before surgery were more likely to be afflicted with severe dysbacteriosis after surgery, and preoperative dysbacteriosis was identified to be predictive of early postoperative diarrhoea. A significantly higher rate of infectious complications was observed in patients with preoperative dysbacteriosis. Moreover, logistic regression revealed that early postoperative diarrhoea and preoperative dysbacteriosis grade 3 were negatively correlated with postoperative complications in rectal cancer patients. According to our interaction analysis**,** a possible explanation was that diarrhoea may act as an intermediate factor between dysbacteriosis and postoperative complications. Diarrhoea, one of the most common symptoms after colectomy with reported incidences from 10 to 31% [37, 38], may render the host susceptible to infections [39]. It has been reported that no specific pathogens, but instead gut dysbiosis, were associated with diarrhoea after organ transplantation [40]. The gut origin of sepsis hypothesis proposes that some of the postoperative infectious complications are due to translocation of pathogens across the intestinal barrier. Diarrhoea was correlated with increased mucosal permeability [41], which further facilitated the translocation of intestinal endogenous bacteria and the absorption of endotoxin [42]. Furthermore, starting supplementation with probiotics [1] or synbiotics [3] 6–7 days before colorectal surgery could protect the mucosal barrier, and lower the rate of diarrhoea and infectious complications, which was in agreement with our findings. However, in the subgroup analysis, early postoperative diarrhoea was not identified as a risk factor for complications after colon resection, which may be related to the degree of preoperative dysbacteriosis in colon cancer patients being less than that in rectal cancer patients. Future studies are needed to clarify the causal effects of dysbacteriosis on early postoperative diarrhoea and infectious complications. Recently, Kok et al. [43] reported that higher dietary fiber intake was associated with a lower risk of surgical complications after CRC resection, the intestinal microbiota involved in fermenting dietary fiber and maintaining mucosal barrier might mediate the observed associations. This finding further promotes that protecting preoperative gut health may be considered in future prehabilitation programmes for CRC patients.

Diarrhoea has also been reported as a potential risk for anastomotic leakage in recent years. Hidaka et al. [26] demonstrated that a faecal volume over 118 ml within 3 days after colorectal surgery may be a reliable predictor for anastomotic leakage. Li et al. [44] found that the incidence of anastomotic leakage was higher in patients with early postoperative diarrhoea after rectal cancer surgery than in those without (16.2% vs. 5.2%, *P* < 0.05). The underlying mechanisms might be that diarrhoea enhances bowel contraction and intraluminal pressure [44], and it is further correlated with microbial dysbacteriosis. Van et al. [45] provided strong evidence that patients with a lack of microbial diversity or with mucin-degrading bacteria in the mucosa at the anastomosis were more likely to develop anastomotic leakage. To date, in our study, anastomotic leakage was also detected at a higher rate in patients with grade 3 dysbacteriosis (Table 3). However, due to the low anastomotic leakage rate and insufficient sample size, we did not exclude the possibility of preoperative dysbacteriosis as an independent risk factor for anastomotic leakage. Bakker et al. [46] reported a higher rate of leakage without giving clear diagnostic evaluation criteria. In our study, although 52 patients with mild symptoms were all evaluated with CT scans, symptomatic leaks might not be counted. Thus, further study is needed to investigate the mechanism between the imbalance of the luminal microbiome and anastomotic complications.

Some of the widely used approaches to characterizing the intestinal microbiota are metagenomics, DNA fingerprinting techniques, and culture. However, owing to their high expenses and inconsistent results, incorporating these techniques into clinical diagnosis and management remains far-off prospect. Gram staining is one of the simplest, most commonly performed, and economical methods for the rapid diagnosis of bacterial and fungal infections [47]. It has been widely used for identifying bacteria in faecal, sputum, urine, or vaginal secretions [48–50], to provide early diagnostic and therapeutic information for the control of bacterial infections [51]. Wu et al. [52] evaluated the accuracy of real-time PCR, gram stain and culture in detecting *S. pneumoniae*, *N. meningitidis*, and *H. influenzae* in 451 cerebrospinal fluid specimens. Gram staining showed high sensitivity (98.2%) and specificity (98.7%) when the culture was positive, while the sensitivity and specificity of real-time PCR were 95.7% and 94.3%, respectively. Consistent with our study, Shimizu et al. [50] reported that Gram-stained faecal flora can be classified into three patterns (diverse pattern, single pattern, and depleted pattern), which were associated with cultured bacterial counts. In addition, the incidence of bacteremia (71% vs. 35%, *P* < 0.05) and mortality due to multiple organ dysfunction syndrome (52% vs. 6%, *P* < 0.05) for single pattern was significantly higher than that for diverse pattern in ICU patients. The classification of intestinal dysbacteriosis in our study was performed according to criteria set up by a Chinese monograph published in 2000 based on the tests of 2349 faecal samples by the Southern Hospital of the First Military Medical University of China [23]. This criterion has been widely adopted by most hospitals, as well as by our hospital. Although it is not able to quantify each bacterial species, this test does reflect the severity of alteration in gut flora [50]. Overall, gram-stained bacteria have the potential to be used as a quick bedside diagnostic marker for infectious complications.

One limitation of our study is that we only considered stool examination, while the distribution of bacteria on the intestinal mucosa biopsy was different from that of faecal bacteria [53]. The conclusion drawn from our study only represents faecal flora. In addition, the quality and quantity of gram staining smears were based on the experience and knowledge of those conducting the tests [48], and a risk of misdiagnosis may be present. However, the bias seems to be acceptable according to our assessment of the stability of repeated tests. Furthermore, we did not record postoperative hyperglycaemia, but a postoperative blood glucose level > 150 mg/dL has been identified as a risk factor for surgical site infections [54], which should be further taken into consideration.

## Conclusion

Intestinal dysbacteriosis has already existed in nearly a quarter of CRC patients before surgery. The severity of bacterial dysbiosis after surgery increases along with the degree of bacterial dysbiosis before surgery. This study indicates preoperative dysbacteriosis as an independent risk factor for postoperative diarrhoea, which further correlates with postoperative infectious complications and anastomotic leakage. However, the contribution of preoperative dysbacteriosis to the occurrence of anastomotic leakage needs to be clarified in further studies.

## Supplementary Information

**Additional file 1.** **Table 1.** Univariate analysis of postoperative infectious complications. **Table 2.** Univariate analysis of early postoperative diarrhoea. **Table 3.** Clinical variables of colon vs. rectal cancer. **Table 4.** Subgroup logistic regression of postoperative diarrhoea.

## Data Availability

The datasets used and/or analysed during the current study are available from the corresponding author on reasonable request.

## Abbreviations

ASA: American Society of Anaesthesiologists
BMI: Body mass index
CDC: Centres for disease control
CDT: Cytolethal distending toxin
CI: Confidence interval
CRC: Colorectal cancer
CRF: Case report form
CT: Computed tomography
ERAS: Enhanced recovery after surgery
IBD: Inflammatory bowel disease
ICD-10: International classification of diseases, 10th edition
OR: Odds ratio
SSIs: Surgical site infections
TNM: Tumour-node- metastasis
