# Supplementary material for: The effects of preoperative intestinal dysbacteriosis on postoperative recovery in colorectal cancer surgery: a prospective cohort study
Source: BMC Gastroenterol. 2021 Nov 25;21:446. doi: 10.1186/s12876-021-02035-6 (PMC8620658; doi:10.1186/s12876-021-02035-6)
Supplement: Supplementary file 1 — Additional file 1. Table 1. Univariate analysis of postoperative infectious complications. Table 2. Univariate analysis of early postoperative diarrhoea. Table 3. Clinical variables of colon vs. rectal cancer. Table 4. Subgroup logistic regression of postoperative diarrhoea. [file 12876_2021_2035_MOESM1_ESM.docx]

**Electronic supplementary material**

**Supplementary Table 1 Univariate analysis of postoperative infectious complications**

|  | Counts  (n=349) | Infectious complications  [N (%)] | $x^{2}$ | *P* |
| --- | --- | --- | --- | --- |
| Sex |  |  | 3.247 | 0.072 |
| Male | 148 | 15 (10.1%) |  |  |
| Female | 201 | 34 (16.9%) |  |  |
| Age (year) |  |  | 0.054 | 0.817 |
| < 75 | 282 | 39 (13.8%) |  |  |
| ≥ 75 | 67 | 10 (14.9%) |  |  |
| BMI (kg/m^2^) |  |  | 0.461 | 0.691 |
| Underweight (< 18.5) | 24 | 2 (8.3%) |  |  |
| Normal (18.5-24.9) | 242 | 34 (14.0%) |  |  |
| Overweight (25.0-29.9) | 80 | 13 (16.3%) |  |  |
| Obese (≥ 30.0) | 3 | 0 (0.0%) |  |  |
| Tumor location |  |  | 0.308 | 0.579 |
| Rectum | 201 | 30 (14.9%) |  |  |
| Colon | 148 | 19 (12.8%) |  |  |
| ASA |  |  | 1.119 | 0.773 |
| 1 | 7 | 1 (14.3%) |  |  |
| 2 | 267 | 35 (13.1%) |  |  |
| 3 | 74 | 13 (17.6%) |  |  |
| 4 | 1 | 0 (0.0%) |  |  |
| TNM stage |  |  | 2.477 | 0.479 |
| I | 67 | 8 (11.9%) |  |  |
| II | 116 | 21 (18.1%) |  |  |
| III | 146 | 17 (11.6%) |  |  |
| IV | 20 | 3 (15.0%) |  |  |
| Comorbidities |  |  | 0.439 | 0.508 |
| No | 177 | 27 (15.3%) |  |  |
| Yes | 172 | 22 (12.8%) |  |  |
| Cardiopulmonary disease | 39 | 5 (12.8%) | 0.054 | 0.816 |
| Diabetes | 25 | 4 (16.0%) | 0.083 | 0.773 |
| Liver or kidney disease | 12 | 2 (16.7%) | 0.071 | 0.790 |
| Anaemia | 134 | 16 (11.9%) | 0.795 | 0.373 |
| Preoperative Albumin (g/L) |  |  | 0.635 | 0.426 |
| < 35 | 17 | 4 (23.5%) |  |  |
| ≥ 35 | 332 | 45 (13.6%) |  |  |
| Postoperative Albumin (g/L) |  |  | 6.782 | 0.009 |
| < 30 | 40 | 11 (27.5%) |  |  |
| ≥ 30 | 309 | 38 (12.3%) |  |  |
| Neoadjuvant therapy |  |  | 0.005 | 0.944 |
| Yes | 77 | 11 (14.3%) |  |  |
| No | 272 | 38 (14.0%) |  |  |
| Preoperative dysbacteriosis |  |  | 11.337 | 0.003 |
| Grade 1 | 265 | 28 (10.6%) |  |  |
| Grade 2 | 57 | 12 (21.1%) |  |  |
| Grade 3 | 27 | 9 (33.3%) |  |  |
| Surgical approach |  |  | 0.146 | 0.702 |
| Open abdominal | 85 | 13 (15.3%) |  |  |
| Laparoscopic | 264 | 36 (13.6%) |  |  |
| Intraoperative blood loss (ml) |  |  | 3.020 | 0.082 |
| < 100 | 275 | 34 (12.4%) |  |  |
| ≥ 100 | 74 | 15 (20.3%) |  |  |
| Operation time (h) |  |  | 1.661 | 0.197 |
| < 3 | 98 | 10 (10.2%) |  |  |
| ≥ 3 | 251 | 39 (15.5%) |  |  |
| Postoperative dysbacteriosis |  |  | 4.811 | 0.090 |
| Grade 1 | 57 | 9 (15.8%) |  |  |
| Grade 2 | 42 | 10 (23.8%) |  |  |
| Grade 3 | 30 | 11 (36.7%) |  |  |
| Early postoperative diarrhoea |  |  | 17.199 | 0.001 |
| Yes | 72 | 21 (29.2%) |  |  |
| No | 277 | 28 (10.1%) |  |  |

**Supplementary Table 2 Univariate analysis of early postoperative diarrhoea**

|  | Counts  (n=353) | Early Postoperative Diarrhoea [N (%)] | $x^{2}$/Z | *P* |
| --- | --- | --- | --- | --- |
| Sex |  |  | 0.115 | 0.735 |
| Male | 150 | 31 (20.7%) |  |  |
| Female | 203 | 45 (22.2%) |  |  |
| Age (year) |  |  | 1.279 | 0.258 |
| < 75 | 286 | 65 (22.7%) |  |  |
| ≥ 75 | 67 | 11 (16.4%) |  |  |
| BMI (kg/m^2^) |  |  | 2.248 | 0.025 |
| Underweight (< 18.5) | 24 | 3 (12.5%) |  |  |
| Normal (18.5-24.9) | 246 | 48 (19.5%) |  |  |
| Overweight (25.0-29.9) | 80 | 25 (31.3%) |  |  |
| Obese (≥ 30.0) | 3 | 0 (0.0%) |  |  |
| Tumor location |  |  | 1.144 | 0.285 |
| Rectum | 204 | 48 (23.5%) |  |  |
| Colon | 149 | 28 (18.8%) |  |  |
| TNM stage |  |  | -0.554 | 0.580 |
| I | 67 | 18 (26.9%) |  |  |
| II | 118 | 24 (20.3%) |  |  |
| III | 147 | 27 (18.4%) |  |  |
| IV | 21 | 7 (33.3%) |  |  |
| Comorbidities |  |  | 0.007 | 0.933 |
| No | 178 | 38 (21.3%) |  |  |
| Yes | 175 | 38 (21.7%) |  |  |
| Cardiopulmonary disease | 39 | 4 (10.3%) | 3.298 | 0.069 |
| Diabetes | 26 | 6 (23.1%) | 0.040 | 0.842 |
| Liver or kidney disease | 12 | 3 (25.0%) | 0.089 | 0.766 |
| Anaemia | 136 | 31 (22.8%) | 0.209 | 0.647 |
| Neoadjuvant therapy |  |  | 1.923 | 0.166 |
| Yes | 77 | 21 (27.3%) |  |  |
| No | 276 | 55 (19.9%) |  |  |
| Preoperative dysbacteriosis |  |  | 35.873 | 0.000 |
| Grade 1 | 268 | 38 (14.2%) |  |  |
| Grade 2 | 58 | 25 (43.1%) |  |  |
| Grade 3 | 27 | 13 (48.1%) |  |  |
| Surgical approach |  |  | 3.863 | 0.049 |
| Open abdominal | 86 | 12 (14.0%) |  |  |
| Laparoscopic | 267 | 64 (24.0%) |  |  |
| Intraoperative blood loss (ml) |  |  | 5.789 | 0.016 |
| < 100 | 277 | 52 (18.8%) |  |  |
| ≥ 100 | 76 | 24 (31.6%) |  |  |
| Operation time (h) |  |  | 0.529 | 0.467 |
| < 3 | 100 | 19 (19.0%) |  |  |
| ≥ 3 | 253 | 57 (22.5%) |  |  |
| Postoperative dysbacteriosis |  |  | 14.504 | 0.001 |
| Grade 1 | 59 | 15 (25.4%) |  |  |
| Grade 2 | 43 | 27 (62.8%) |  |  |
| Grade 3 | 30 | 14 (46.7%) |  |  |
| Mechanical bowel preparation |  |  | 0.602 | 0.438 |
| Yes | 214 | 49 (22.9%) |  |  |
| No | 139 | 27 (19.4%) |  |  |
| Antibiotics (days) |  |  | 33.106 | <0.001 |
| ≤ 3 | 294 | 47 (15.9%) |  |  |
| >3 | 58 | 29 (50.0%) |  |  |

**Supplementary Table 3 Clinical variables of colon vs. rectal cancer**

| Variables | Total  (n=353) | Rectal cancer  (n=204) | Colon cancer  (n=149) | $x^{2}$ | *P* |
| --- | --- | --- | --- | --- | --- |
| Sex |  |  |  | 1.536 | 0.215 |
| Male | 150 (42.5%) | 81 (39.7%) | 69 (46.3%) |  |  |
| Female | 203 (57.5%) | 123 (60.3%) | 80 (53.7%) |  |  |
| Age (year) |  |  |  | 0.813 | 0.367 |
| < 75 | 286 (81.0%) | 162 (79.4%) | 124 (83.2%) |  |  |
| ≥ 75 | 67 (19.0%) | 42 (20.6%) | 25 (16.8%) |  |  |
| BMI (kg/m^2^) |  |  |  | 11.036 | 0.012 |
| Underweight (< 18.5) | 24 (6.8%) | 7 (3.4%) | 17 (11.4%) |  |  |
| Normal (18.5-24.9) | 246 (69.7%) | 144 (70.6%) | 102 (68.5%) |  |  |
| Overweight (25.0-29.9) | 80 (22.7%) | 50 (24.5%) | 30 (20.1%) |  |  |
| Obese (≥ 30.0) | 3 (0.8%) | 3 (1.5%) | 0 (0%) |  |  |
| Bowel preparation |  |  |  | 113.629 | <0.001 |
| Yes | 214 (60.6%) | 172 (84.3%) | 42 (28.2%) |  |  |
| No | 139 (39.4%) | 32 (15.7%) | 107 (71.8%) |  |  |
| ASA |  |  |  | 2.096 | 0.553 |
| 1 | 7 (2.0%) | 3 (1.5%) | 4 (2.7%) |  |  |
| 2 | 271 (76.8%) | 157 (77.0%) | 114 (76.5%) |  |  |
| 3 | 74 (21.0%) | 44 (21.6%) | 30 (20.1%) |  |  |
| 4 | 1 (0.3%) | 0 (0%) | 1 (0.7%) |  |  |
| TNM stage |  |  |  | 18.714 | <0.001 |
| I | 67 (19.0%) | 54 (26.5%) | 13 (8.7%) |  |  |
| II | 118 (33.4%) | 63 (30.9%) | 55 (36.9%) |  |  |
| III | 147 (41.6%) | 74 (36.3%) | 73 (49.0%) |  |  |
| IV | 21 (5.9%) | 13 (6.4%) | 8 (5.4%) |  |  |
| Comorbidities |  |  |  |  |  |
| Cardiopulmonary disease | 39 (11.0%) | 18 (8.8%) | 21 (14.1%) | 2.434 | 0.119 |
| Diabetes | 26 (7.4%) | 11 (5.4%) | 15 (10.1%) | 2.758 | 0.097 |
| Liver or kidney disease | 12 (3.4%) | 7 (3.4%) | 5 (3.4%) | 0.002 | 0.969 |
| Preoperative anaemia | 136 (38.5%) | 65 (31.9%) | 71 (47.7%) | 9.063 | 0.003 |
| Preoperative Albumin (g/L) |  |  |  | 2.778 | 0.096 |
| < 35 | 18 (5.1%) | 7 (3.4%) | 11 (7.4%) |  |  |
| ≥ 35 | 335 (94.9%) | 197 (96.6%) | 138 (92.6%) |  |  |
| Postoperative Albumin (g/L) |  |  |  | 1.186 | 0.276 |
| < 30 | 42 (11.9%) | 21 (10.3%) | 21 (14.1%) |  |  |
| ≥ 30 | 311 (88.1%) | 183 (89.7%) | 128 (85.9%) |  |  |
| Neoadjuvant therapy |  |  |  | 16.363 | <0.001 |
| Yes | 77 (21.8%) | 60 (29.4%) | 17 (11.4%) |  |  |
| No | 276 (78.2%) | 144 (70.6%) | 132 (88.6%) |  |  |
| Preoperative dysbacteriosis |  |  |  | 7.282 | 0.026 |
| Grade 1 | 268 (75.9%) | 160 (78.4%) | 108 (72.5%) |  |  |
| Grade 2 | 58 (16.4%) | 25 (12.3%) | 33 (22.1%) |  |  |
| Grade 3 | 27 (7.6%) | 19 (9.3%) | 8 (5.4%) |  |  |
| Surgical approach |  |  |  | 2.829 | 0.093 |
| Open abdominal | 86 (24.4%) | 43 (21.1%) | 43 (28.9%) |  |  |
| Laparoscopic | 267 (75.6%) | 161 (78.9%) | 106 (71.1%) |  |  |
| Intraoperative blood loss (ml) |  |  |  | 0.297 | 0.586 |
| < 100 | 277 (78.5%) | 158 (77.5%) | 119 (79.9%) |  |  |
| ≥ 100 | 76 (21.5%) | 46 (22.5%) | 30 (20.1%) |  |  |
| Operation time (h) |  |  |  | 6.660 | 0.010 |
| < 3 | 100 (28.3%) | 47 (23.0%) | 53 (35.6%) |  |  |
| ≥ 3 | 253 (71.7%) | 157 (77.0%) | 96 (64.4%) |  |  |
| Postoperative dysbacteriosis |  |  |  | 4.804 | 0.091 |
| Grade 1 | 59 (44.7%) | 31 (43.7%) | 28 (45.9%) |  |  |
| Grade 2 | 43 (32.6%) | 19 (26.8%) | 24 (39.3%) |  |  |
| Grade 3 | 30 (22.7%) | 21 (29.6%) | 9 (14.8%) |  |  |
| Early postoperative diarrhoea | 76 (21.5%) | 48 (23.5%) | 28 (18.8%) | 1.144 | 0.285 |
| Surgical site infections | 24 (6.8%) | 16 (7.8%) | 8 (5.4%) | 0.832 | 0.362 |
| Incision infections | 8 (2.3%) | 4 (2.0%) | 4 (2.7%) | 0.204 | 0.652 |
| Abdominal/Pelvic infections | 10 (2.8%) | 8 (3.9%) | 2 (1.3%) | 2.081 | 0.149 |
| Anastomotic leakage | 10 (2.8%) | 8 (3.9%) | 2 (1.3%) | 2.081 | 0.149 |
| Pulmonary infections | 29 (8.2%) | 19 (9.3%) | 10 (6.7%) | 0.773 | 0.379 |
| Urinary tract infections | 4 (1.1%) | 1 (0.5%) | 3 (2.0%) | 1.783 | 0.182 |
| Fever of unknown origin | 3 (0.8%) | 2 (1.0%) | 1 (0.7%) | 0.098 | 0.755 |
| Total infectious complications | 53 (15.0%) | 33 (16.2%) | 20 (13.4%) | 0.512 | 0.474 |
| Length of postoperative stay (days), *median (range)* | 6.0 (3-32) | 6.0 (3-32) | 5.0 (3-25) | -6.887 | <0.001 |
| Length of antibiotic therapy (days), *median (range)* | 2.0 (1-24) | 2.0 (1-24) | 2.0 (1-20) | -0.720 | 0.472 |

**Supplementary Table 4 Subgroup logistic regression of postoperative diarrhoea**

| Parameter | Univariate logistic regression | | Multivariate logistic regression ^1^ | |
| --- | --- | --- | --- | --- |
|  | OR (95% CI) | *P* | Adjusted OR (95% CI) | *P* |
| **Colon cancer** |  |  |  |  |
| Preoperative dysbacteriosis |  |  |  |  |
| Grade 2 | 3.10 (1.25-7.67) | 0.014 | 3.89 (1.41-10.74) | 0.009 |
| Grade 3 | 2.07 (0.38-11.21) | 0.400 | 3.13 (0.53-18.52) | 0.209 |
| Neoadjuvant therapy | 2.73 (0.91-8.15) | 0.073 | 3.04 (0.90-10.27) | 0.073 |
| BMI (≥25.0 kg/m^2^) | 2.26 (0.90-5.67) | 0.084 | 3.11 (1.11-8.73) | 0.031 |
| Length of antibiotics therapy >3 days | 3.59 (1.36-9.46) | 0.010 | 2.98 (1.05-8.46) | 0.040 |
| **Rectal cancer** |  |  |  |  |
| Preoperative dysbacteriosis |  |  |  |  |
| Grade 2 | 7.58 (3.07-18.74) | 0.001 | 6.29 (2.43-16.33) | <0.001 |
| Grade 3 | 8.19 (2.98-22.54) | 0.001 | 5.51 (1.81-16.82) | 0.003 |
| Intraoperative blood loss (≥100 ml) | 3.57 (1.75-7.28) | <0.001 | 2.76 (1.23-6.19) | 0.014 |
| Length of antibiotics therapy >3 days | 6.71 (3.07-14.69) | <0.001 | 4.31 (1.82-10.19) | 0.001 |

^1^ Hosmer-Lemeshow test:${colon cancer x}^{2}$=2.690, *P*=0.611, rectal cancer$x^{2}$=3.077, *P*=0.380.
